# Urine hepcidin has additive value in ruling out cardiopulmonary bypass-associated acute kidney injury: an observational cohort study

**DOI:** 10.1186/cc10339

**Published:** 2011-08-04

**Authors:** Anja Haase-Fielitz, Peter R Mertens, Michael Plaß, Hermann Kuppe, Roland Hetzer, Mark Westerman, Vaughn Ostland, John R Prowle, Rinaldo Bellomo, Michael Haase

**Affiliations:** 1 Department of Nephrology and Hypertension & Endocrinology and Metabolic Diseases, Otto-von-Guericke-University, Leipziger Strasse 44, D-39120 Magdeburg, Germany; 2 Institute of Anesthesiology, German Heart Center, Augustenburger Platz 1, D-13353 Berlin, Germany; 3 Department of Cardiothoracic Surgery, German Heart Center, Augustenburger Platz 1, D-13353 Berlin, Germany; 4 Intrinsic LifeSciences LLC, 505 Coast Boulevard South, Suite 102, La Jolla, CA 92037, USA; 5 Department of Intensive Care, Austin Hospital, Studley Road, Heidelberg 3084, Melbourne, Australia

## Abstract

**Introduction:**

Conventional markers of acute kidney injury (AKI) lack diagnostic accuracy and are expressed only late after cardiac surgery with cardiopulmonary bypass (CPB). Recently, interest has focused on hepcidin, a regulator of iron homeostasis, as a unique renal biomarker.

**Methods:**

We studied 100 adult patients in the control arm of a randomized, controlled trial http://www.clinicaltrials.gov/NCT00672334 who were identified as being at increased risk of AKI after cardiac surgery with CPB. AKI was defined according to the Risk, Injury, Failure, Loss, End-stage renal disease classification of AKI classification stage. Samples of plasma and urine were obtained simultaneously (1) before CPB (2) six hours after the start of CPB and (3) twenty-four hours after CPB. Plasma and urine hepcidin 25-isoforms were quantified by competitive enzyme-linked immunoassay.

**Results:**

In AKI-free patients (*N *= 91), urine hepcidin concentrations had largely increased at six and twenty-four hours after CPB, and they were three to seven times higher compared to patients with subsequent AKI (*N *= 9) in whom postoperative urine hepcidin remained at preoperative levels (*P *= 0.004, *P *= 0.002). Furthermore, higher urine hepcidin and, even more so, urine hepcidin adjusted to urine creatinine at six hours after CPB discriminated patients who did not develop AKI (area under the curve (AUC) receiver operating characteristic curve 0.80 [95% confidence interval (95% CI) 0.71 to 0.87] and 0.88 [95% CI 0.78 to 0.97]) or did not need renal replacement therapy initiation (AUC 0.81 [95% CI 0.72 to 0.88] 0.88 [95% CI 0.70 to 0.99]) from those who did. At six hours, urine hepcidin adjusted to urine creatinine was an independent predictor of ruling out AKI (*P *= 0.011). Plasma hepcidin did not predict no development of AKI. The study findings remained essentially unchanged after excluding patients with preoperative chronic kidney disease.

**Conclusions:**

Our findings suggest that urine hepcidin is an early predictive biomarker of ruling out AKI after CPB, thereby contributing to early patient risk stratification.

## Introduction

Acute kidney injury (AKI) associated with cardiopulmonary bypass (CPB) is relatively common and carries significantly increased morbidity, mortality and cost [1]. It is now the second most common cause of severe AKI in critically ill patients [2]. Its pathogenesis is complex [3], and the development of targeted new treatments is likely to depend on early diagnosis. Unfortunately, conventional markers of AKI, such as serum creatinine, serum urea and urine output, lack diagnostic sensitivity and specificity and often identify patients with AKI only 24 or 48 hours after surgery [4-6]. Accordingly, progress in this field is likely to depend, at least in part, on the availability of novel biomarkers for early and reliable diagnosis of AKI.

Over the past decade a number of early markers of AKI have been identified using genomic [7,8] and proteomic [9] laboratory methods. These biomarkers may prove useful in the early identification of patients at risk [5,6]. More recently, interest has focused on hepcidin as a regulator of iron homeostasis [10]. The hepcidin gene is regulated by iron loading, hypoxia or inflammation [10-12], and its protein is produced by liver and kidney cells [13]. In a pilot study of a nested cohort of 22 patients with AKI and 22 without AKI, Ho *et al. *[14] reported a greater postoperative signal/noise ratio for urine hepcidin levels in those patients who did not go on to develop AKI, suggesting that hepcidin may be the first clinically useful negative biomarker for AKI after CPB.

Accordingly, we investigated the association of urine and plasma hepcidin levels in patients undergoing cardiac surgery with the use of CPB. We aimed to (1) assess the predictive value of early postoperative urine hepcidin and plasma hepcidin for ruling out AKI, (2) investigate the role of chronic kidney disease (CKD) on the predictive value of hepcidin and (3) explore whether changes in urine hepcidin reflect changes in plasma hepcidin.

## Materials and methods

### Patient population

For the purpose of our study, we investigated all 100 adult patients of the German study site (German Heart Center Berlin) enrolled into the control arm of the Sodium Bicarbonate in Cardiac Surgery Study http://www.clinicaltrials.gov/NCT00672334, a multicenter, randomized, controlled trial of perioperative sodium bicarbonate versus placebo for the prevention of AKI and the exploration of renal biomarkers in patients at increased renal risk undergoing cardiac surgery necessitating the use of CPB. Increased risk of AKI was defined as at least one risk factor for postoperative AKI: age ≥70 years; New York Heart Association class III/IV or left ventricular ejection fraction (LVEF) <35%; insulin-depending diabetes mellitus; prior cardiac surgery; valvular surgery (with or without coronary artery bypass graft) or preoperative serum creatinine >120 μmol/L.

We excluded patients undergoing emergency operations (time between hospital admission to operation ≤24 hours) or off-pump surgery, patients presenting with advanced CKD (serum creatinine >300 μmol/L) or kidney transplant and patients <18 years old. Clinical practice was not changed or modified for the purpose of the study.

CKD was defined as preoperative estimated glomerular filtration rate (eGFR) <60 mL/minute/1.73 m^2 ^[15]. eGFR was estimated using the Modification of Diet in Renal Disease Study equation reexpressed for use with the serum creatinine values standardized to isotope dilution mass spectroscopy [16]. Renal replacement therapy (RRT) was initiated if the patient fulfilled at least one of the following clinical criteria: oliguria (urine output <100 mL/>6 hours) that was unresponsive to fluid resuscitation measures, hyperkalemia (potassium >6.5 mmol/L), severe acidosis (pH <7.2) or clinically significant organ edema (for example, lung) in the setting of renal failure.

Samples were obtained from 100 patients enrolled in the placebo group (sodium chloride 1.2 L starting with anesthesia induction and finishing 24 hours thereafter). Patients were recruited between January 2009 and June 2010. The local Institutional Review Board approved this investigation, and written, informed consent was obtained from each patient, including the investigation of novel renal biomarkers. The study was carried out in compliance with the Declaration of Helsinki.

### Sampling

Samples of plasma and urine were obtained simultaneously (1) directly after insertion of an arterial line (baseline) before induction of anesthesia, (2) at 6 hours after commencement of CPB and (3) at 24 hours after commencement of CPB. The timing of sampling was chosen to detect renal biomarkers before serum creatinine increases and was therefore limited to the first 24 hours postoperatively. Aliquots of plasma and urine were frozen and stored at -80°C immediately after collection and centrifugation and were kept frozen on dry ice during transport.

### Data collection and outcome definition

Demographic and clinical data (Table 1) were collected at baseline and for the first 48 hours. Serum creatinine was measured at baseline, at 6 and 24 hours after commencement of CPB, daily within the first postoperative week and, if required, until hospital discharge. The primary outcome, AKI, was defined based on the baseline-to-peak serum creatinine increase (>50%) or urine output decrease (<0.5 ml/kg/hr for at least 6 hours) during the first seven postoperative days using the Risk, Injury, Failure, Loss, End-stage renal disease classification of AKI (RIFLE) consensus definition of AKI [17,18]. Additional analyses were performed to assess the ability of hepcidin to predict the severity of AKI using RIFLE classes R, I and F and the need for RRT initiation. The Strengthening the Reporting of Observational Studies in Epidemiology guidelines [19] for reporting observational studies were used.

**Table 1:** Characteristics of patients developing AKI compared with those who did not develop AKI

	All Patients			Excluding Patients With Preoperative CKD		
		
Variables	AKI *N *= 9	No AKI *N *= 91	*P *value	AKI *N *= 5	No AKI *N *= 69	*P *value
Age, y	74 (70-77)	67 (56-73)	0.015	75 (73-84)	65 (55-72)	0.003
Female, n	2 (22.2%)	31 (34.1%)	0.47	1 (20.0%)	19 (27.5%)	>0.99
Preoperative eGFR, mL/min/1.73 m^2^	61 (52-91)	74 (62-88)	0.58	86 (72-100)	80 (70-92)	0.52
Preoperative CKD, n	4 (44.5%)	22 (24.2%)	0.23	-	-	-
LVEF <35%, n	3 (33.3%)	8 (8.8%)	0.06	1 (20.0%)	6 (8.7%)	0.40
Atrial fibrillation	6 (66.7%)	25 (27.5%)	0.015	4 (80.0%)	18 (26.1%)	0.025
Arterial hypertension	8 (88.9%)	65 (71.4%)	0.26	5 (100.0%)	48 (69.6%)	0.31
COPD	3 (33.3%)	8 (8.8%)	0.025	2 (40.0%)	6 (8.7%)	0.087
Diabetes mellitus, n	3 (33.3%)	17 (18.7%)	0.38	0 (0.0%)	12 (17.4%)	0.58
PVD, n	4 (44.5%)	17 (18.7%)	0.09	2 (40.0%)	11 (15.9%)	0.21

AKI, acute kidney disease; AKI defined as RIFLE class R or worse including serum creatinine increase and urine output decrease.

Diabetes mellitus defined as diabetes on medication (insulin or oral antidiabetics).

CKD, chronic kidney disease; COPD, chronic obstructive pulmonary disease; eGFR, estimated glomerular filtration rate; LVEF, left ventricular ejection fraction;

PVD, peripheral vascular disease.

Median (25^th ^to 75^th ^percentiles).

^a^AKI, acute kidney injury; CKD, chronic kidney disease; COPD, chronic obstructive pulmonary disease; eGFR, estimated glomerular filtration rate; LVEF, left ventricular ejection fraction; PVD, peripheral vascular disease. AKI is defined as Risk, Injury, Failure, Loss, End-stage renal disease classification of AKI (RIFLE) class R or worse, including serum creatinine increase and urine output decrease. Diabetes mellitus is defined as patients with diabetes on medication (insulin or oral antidiabetics). Data are medians (25th to 75th percentiles) unless otherwise indicated.

### Biochemical analysis

Coinvestigators performing hepcidin assays were blinded to patient details and AKI classification. Human urine and plasma hepcidin 25-isoforms were measured by competitive enzyme-linked immunoassay, as previously described [20], by Intrinsic LifeSciences LLC (La Jolla, CA, USA). The lower limit of hepcidin detection was 5.5 ng/mL. The median coefficients of variation were 10% for intraassay precision and 6% for interassay reproducibility. Hepcidin stability during sample storage was tested prior to this study with median change in hepcidin concentrations in plasma <5% and in urine <6% after six months at -80°C. For the purposes of this study, samples were assayed after storage at -80°C. Hepcidin values are expressed as nanograms per milliliter. To compensate for perioperative variation in urine dilution, urine hepcidin adjusted to urine creatinine was calculated and expressed as nanograms of hepcidin per milligram of creatinine. Serum creatinine assays were carried out by Austin Health pathology services (Melbourne, Australia) using the modified Jaffé method. Fractional excretion (FE) of hepcidin, that is, the proportion of filtered hepcidin that appears in urine, was calculated using the following formula:

where U_hepcidin _= urine hepcidin concentration, Pl_hepcidin _= plasma hepcidin concentration, Pl_creat _= plasma creatinine concentration and U_creat _= urine creatinine concentration.

### Statistical analysis

Statistical analysis was performed using SPSS version 16.0 software (SPSS Inc., Chicago, IL, USA) and MedCalc 11.5 software (Mariakerke, Belgium). Categorical data were reported as percentages with 95% confidence intervals (95% CIs) of the mean percentage and were compared using Fisher's exact test. After testing for normal distribution, continuous data were reported as medians with 25th to 75th percentiles, and nonparametric data were compared using the Mann-Whitney *U *test. Continuous data over time were compared using the one-way repeated measures analysis of variance by ranks (Friedman test). We used nonparametric bivariate correlation and report Spearman correlation coefficients (*r*). The ability of hepcidin to predict AKI was assessed by plotting receiver operating characteristic (ROC) curves and further reported as areas under the curve (AUC) with 95% CIs. An AUC-ROC value of >0.7 was taken to indicate reasonable biomarker performance and >0.8 was taken to indicate good biomarker performance [21]. An AUC-ROC difference >0.1 units between biomarkers was defined as significant. ROC curve optimal cutoff values for AKI diagnosis, for curves with a statistically significant AUC, were defined as the point that maximized the Youden index, defined as (sensitivity + specificity) - 1 [22]. The increased discriminative value of the biomarkers regarding the composite end point was studied by assessing the net reclassification improvement (NRI) as described by Pencina *et al. *[23]. Univariate and multivariate stepwise regression analyses were undertaken to assess predictors of no AKI after CPB. Variables tested on univariate relation with incidence of no AKI included clinically relevant variables, all of which are displayed in Table 1 as well as the type and duration of surgery and renal biomarkers at six hours after the start of CPB. Multivariate logistic regression modeling included clinically relevant variables with a univariate *P *value <0.1 (age, atrial fibrillation, LVEF <35%, chronic obstructive pulmonary disease [COPD], peripheral vascular disease [PVD]) and renal biomarkers. Logarithmic transformations were applied when necessary before multivariable logistic regression analyses were performed. Statistical significance was denoted by two-sided *P *values <0.05.

## Results

### Sampling

**Figure 1:**
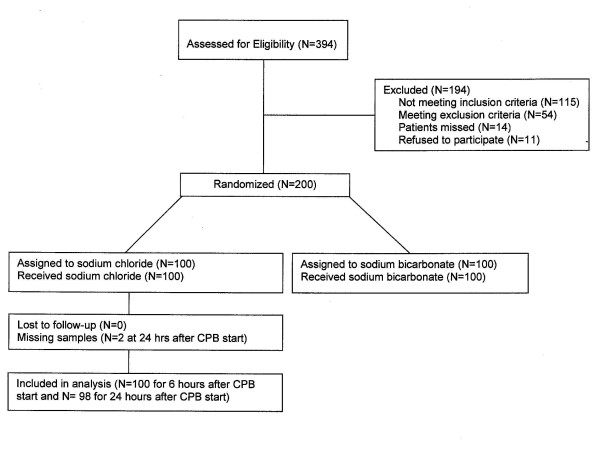
**Patient flow through the study http://www.clinicaltrials.gov/NCT00672334. CPB, cardiopulmonary bypass**.

Figure 1 shows the patient flow through the study. Three hundred ninety-four consecutive patients were screened according to the inclusion and exclusion criteria. Of those, 200 patients were randomized, with all of them undergoing surgery as planned. Of the 100 control patients (analyzed cohort on hepcidin), all had full clinical data sets and complete sampling, except in two patients (no AKI) from whom plasma and urine samples could not be collected at 24 hours after CPB.

### Patient characteristics

Compared with patients with postoperative AKI, AKI-free patients (*n *= 91) were younger and less frequently had preoperative atrial fibrillation and COPD (Table 1). Type and duration of operation did not differ between patients with postoperative AKI and those without, whereas perioperative fluid balance, dose of furosemide and volume of red blood cell transfusion were lower and outcomes were better, including less frequent need for RRT initiation, in AKI-free patients (Table 2). Of patients with AKI, RRT was initiated in three cases, and in those without AKI, RRT was commenced in two patients, in both cases due to anuria for less than six hours and preoperative CKD.

**Table 2:** Interventions and outcomes^a^

	All patients			Excluding patients with preoperative CKD		
Variables	AKI (*n *= 9)	No AKI (*n *= 91)	*P *value	AKI (*n *= 5)	No AKI (*n *= 69)	*P *value
CABG surgery, *n *(%)	2 (22.2%)	17 (18.7%)	0.68	1 (20.0%)	13 (18.8%)	>0.99
Valve surgery, *n *(%)	4 (44.5%)	46 (50.5%)	>0.99	2 (40.0%)	37 (53.6%)	0.66
CABG and valve surgery, *n *(%)	3 (33.3%)	21 (23.1%)	0.68	2 (40.0%)	14 (20.3%)	0.29
Thoracic aortic surgery, *n *(%)	0 (0.0%)	7 (7.7%)	>0.99	0 (0.0%)	5 (7.3%)	>0.99
Previous cardiothoracic operation, *n *(%)	2 (22.2%)	26 (28.6%)	>0.99	1 (20.0%)	23 (33.3%)	>0.99
Duration of bypass, minutes	125 (100 to 172)	119 (91 to 158)	0.62	109 (80 to 146)	119 (90 to 156)	0.57
Fluid balance^b^, mL	4,980 (3,000 to 16,200)	3,100 (1,690 to 4,660)	0.035	4,920 (450 to 16,200)	3,100 (1,260 to 4,800)	0.4
Furosemide^b^, *n *(%)	9 (100%)	84 (92.3%)	0.39	5 (100.0%)	63 (91.3%)	>0.99
Furosemide^b^, mg	130 (60 to 460)	50 (20 to 90)	0.003	160 (35 to 672)	45 (20 to 70)	0.05
Vasopressor use^b^, *n *(%)	7 (77.8%)	65 (71.4%)	0.69	5 (100.0%)	49 (71.0%)	0.32
Inotrope use^b^, *n *(%)	8 (88.9%)	51 (63.7%)	0.13	4 (80.0%)	45 (65.2%)	0.66
Blood transfusion^b^, *n *(%)	8 (88.9%)	52 (57.1%)	0.06	4 (80.0%)	39 (56.5%)	0.39
Blood transfusion^b^, mL	1,000 (500 to 6,380)	500 (0 to 500)	0.005	1,250 (250 to 6,800)	250 (0 to 500)	0.048
LOS in hospital, days	14 (8 to 19)	9 (7 to 14)	0.024	14 (8 to 19)	9 (7 to 14)	0.48
Need for RRT, *n *(%)	3 (33.3%)	2 (2.2%)	0.005	2 (40.0%)	0 (0.0%)	0.004
Hospital mortality, *n *(%)	3 (33.3%)	0 (0%)	0.001	1 (20.0%)	0. (0.0%)	0.068

^a^AKI, acute kidney injury; CKD, chronic kidney disease; CABG, coronary artery bypass graft; LOS, length of stay; RRT, renal replacement therapy. AKI is defined as Risk, Injury, Failure, Loss, End-stage renal disease classification of AKI (RIFLE) class R or worse including serum creatinine increase and urine output decrease. Data are medians (25th to 75th percentiles) unless otherwise indicated. ^b^Including intraoperative and 48-hour postoperative values.

### Urine hepcidin

**Figure 2:**
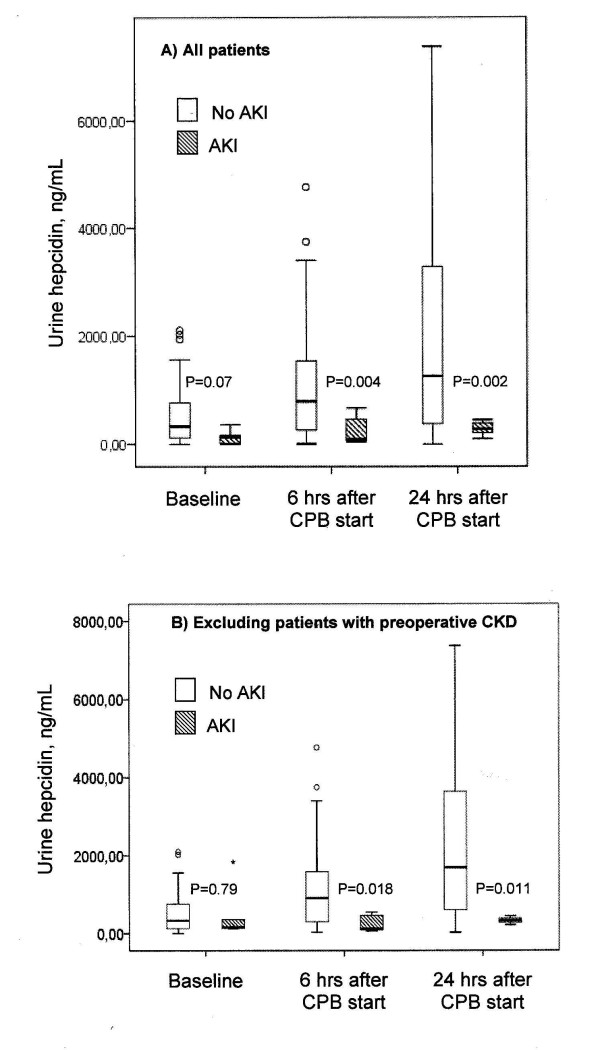
**Urine hepcidin concentration over time**. Patients not developing acute kidney injury (AKI) (white bars) are compared to those with AKI (hatched bars) **(A) **for all patients and **(B) **after excluding patients with chronic kidney disease (CKD). Error bars at top and bottom define th 95% confidence interval and the black lines within boxes the medians.

Starting with a nonsignificant difference at baseline, at six hours and at twenty-four hours after commencement of CPB, urine hepcidin levels were significantly higher in AKI-free patients (Figure 2A). AUC-ROC curves for urine hepcidin at six hours for the discrimination of AKI-free patients from patients with AKI was 0.80 (Figure 4A). Further, the negative predictive value for not developing AKI was 96.3% and the positive predictive value was 31.6%. At six hours, the NRI of urine hepcidin for ruling out AKI was 17% compared to serum creatinine. For the prediction of no RRT initiation, the AUC-ROC curve for urine hepcidin at six hours was 0.81 (Figure 5A). The predictive value at 24 hours remained essentially unchanged (no AKI: AUC-ROC curve 0.81, 95% CI 0.73 to 0.90; no RRT: 0.77, 95% CI 0.64 to 0.89).

When each RIFLE class was considered, the AUC-ROC curves for urine hepcidin at six hours were 0.81 for not developing RIFLE R (*n *= 4) (95% CI 0.71 to 0.88, sensitivity 75%, specificity 86%, cutoff >120 ng/mL), 0.81 for not developing RIFLE I (*n *= 3) (95% CI 0.72 to 0.89, sensitivity 100%, specificity 62%, cutoff >460 ng/mL) and 0.75 for not developing RIFLE F (*n *= 2) (95% CI 0.65 to 0.83, sensitivity 100%, specificity 58%, cutoff >550 ng/mL).

### Urine hepcidin adjusted to urine creatinine

At baseline, urine hepcidin adjusted to urine creatinine (Table 3) tended to be higher in AKI-free patients. At six and twenty-four hours after commencement of CPB, urine hepcidin adjusted to urine creatinine was significantly higher in patients not developing AKI (Table 3). The AUC-ROC curves were 0.88 for predicting an AKI-free status (Figure 4B) and 0.88 for no RRT initiation (Figure 5B). At 24 hours, the AUC-ROC curve declined (no AKI: 0.68, 95% CI 0.50 to 0.87; no RRT: 0.73, 95% CI 0.53 to 0.92).

**Table 3:** Perioperative hepcidin indices^a^

	All patients			Excluding patients with preoperative CKD		
Hepcidin index	No AKI (*n *= 91)	AKI (*n *= 9)	*P *value	No AKI (*n *= 69)	AKI (*n *= 5)	*P *value
Urine hepcidin/urine creatinine, ng/mg						
Baseline	308 (122 to 583)	120 (34 to 295)	0.07	309 (143 to 570)	164 (100 to 633)	0.6
6 hours after CPB start	5,175 (2,086 to 9,539)	1,229 (314 to 2,379)	<0.001	5,402 (2,100 to 9,300)	1,400 (639 to 2,379)	0.009
24 hours after CPB start^b^	3,255 (1,576 to 6,652)	1,345 (537 to 3,583)	0.07	3,143 (1,798 to 6,585)	1,345 (971 to 3,362)	0.06
Friedman test	<0.001	0.005		<0.001	0.11	
Urine creatinine, mg/mL						
Baseline	1.14 (0.57 to 1.74)	0.92 (0.51 to 1.83)	0.52	1.28 (0.60 to 1.96)	1.66 (0.78 to 2.10)	0.71
6 hours after CPB start	0.19 (0.10 to 0.28)	0.21 (0.10 to 0.30)	0.84	0.19 (0.11 to 0.28)	0.21 (0.12 to 0.30)	0.97
24 hours after CPB start^b^	0.46 (0.24 to 0.71)	0.22 (0.12 to 0.29)	0.008	0.52 (0.29 to 0.73)	0.22 (0.18 to 0.37)	0.022
Friedman test	<0.001	0.03		<0.001	0.17	
Urine/plasma hepcidin ratio						
Baseline	2.6 (1.2 to 4.9)	1.1 (0.2 to 5.6)	0.18	2.8 (1.2 to 4.8)	4.6 (1.6 to 8.4)	0.41
6 hours after CPB start	3.1 (1.5 to 5.3)	1.0 (0.2 to 3.1)	0.047	3.6 (1.9 to 5.9)	3.0 (1.0 to 46.7)	0.59
24 hours after CPB start^b^	6.9 (2.0 to 13.3)	1.2 (0.6 to 2.4)	0.011	8.2 (3.8 to 15.1)	2.3 (0.8 to 11.6)	0.11
Friedman test	<0.001	0.69		<0.001	0.74	
Fractional hepcidin excretion, %						
Baseline	2.4 (1.5 to 4.1)	1.7 (0.7 to 2.5)	0.17	2.3 (1.5 to 2.9)	2.5 (1.8 to 4.7)	0.58
6 hours after CPB start	22.1 (12.2 to 30.0)	8.3 (1.7 to 19.5)	0.005	22.0 (15.1 to 30.5)	16.3 (3.6 to 22.5)	0.19
24 hours after CPB start^b^	14.7 (9.5 to 25.2)	8.1 (4.5 to 46.5)	0.35	15.3 (9.9 to 25.2)	8.1 (5.1 to 55.7)	0.6
Friedman test	<0.001	0.021		<0.001	0.47	

^a^AKI, acute kidney injury; CKD, chronic kidney disease; CPB; cardiopulmonary bypass. AKI is defined as Risk, Injury, Failure, Loss, End-stage renal disease classification of AKI (RIFLE) class R or worse including serum creatinine increase and urine output decrease. Data are medians (25th to 75th percentiles) unless otherwise indicated. ^b^Values at 24 hours after CPB start refer to 89 patients without AKI and 9 patients with AKI.

Urine creatinine at six hours after CPB alone did not separate patients with subsequent AKI from those without (Table 3). Changes (from baseline to six hours) in urine hepcidin levels adjusted to urine creatinine did not change the predictive value and did not indicate a carryover effect from baseline to postoperative values, given a different relative change (Table 4).

**Table 4:** Changes in urine hepcidin levels adjusted to urine creatinine^a^

Urine hepcidin/urine creatinine	AKI (*n *= 9)	No AKI (*n *= 91)	*P *value
Δ 6 to 0 hours, ng/mg	1,079 (85 to 2,195)	4,085 (1,998 to 8,736)	<0.001
Δ 6 to 0 hours, %	626 (108 to 1,541)	1,444 (867 to 3,269)	0.016
Δ 24 to 0 hours, ng/mg	1,142 (344 to 3,379)	2,821 (1,266 to 6,482)	0.119
Δ 24 to 0 hours, %	925 (270 to 1,825)	939 (338 to 2,458)	0.717
Δ 24 to 6 hours, ng/mg	325 (-397 to 1,959)	-1,051 (-4,350 to 1,531)	0.079
Δ 24 to 6 hours, %	139 (-29 to 235)	-33 (-71 to 71)	0.02

^a^AKI, acute kidney injury. Data are medians (25th to 75th percentiles). Urine hepcidin/urine creatinine 6 hours to 0 hours for AKI: area under the curve receiver operating characteristic curve 0.88 (0.79 to 0.98) (P < 0.001), sensitivity 75%, specificity 87%, cutoff 1,320 ng/mg.

### Plasma hepcidin

**Figure 3:**
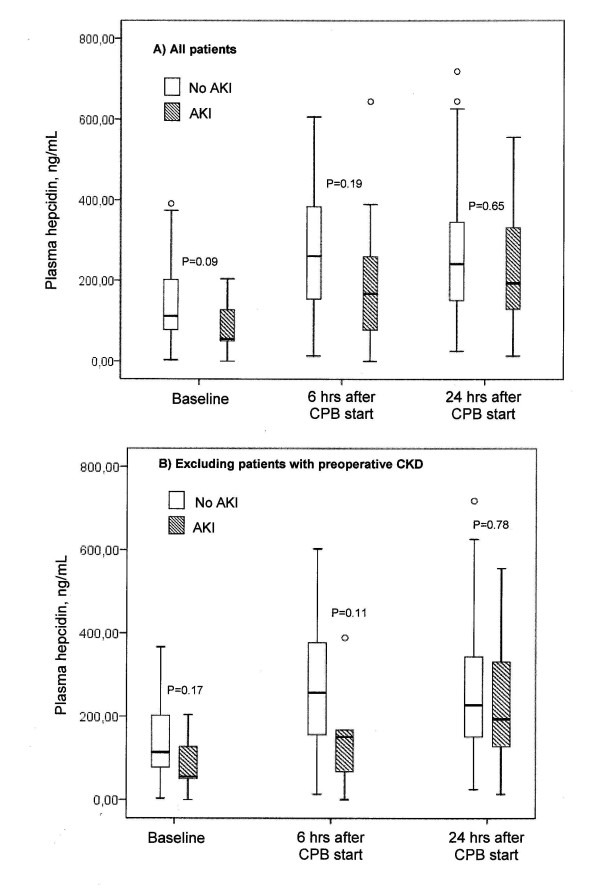
**Plasma hepcidin concentration over time**. Patients not developing AKI (white bars) are compared to those with AKI (hatched bars) **(A) **for all patients and **(B) **after excluding patients with CKD. Error bars at top and bottom define th 95% confidence interval and the black lines within boxes the medians.

**Figure 4:**
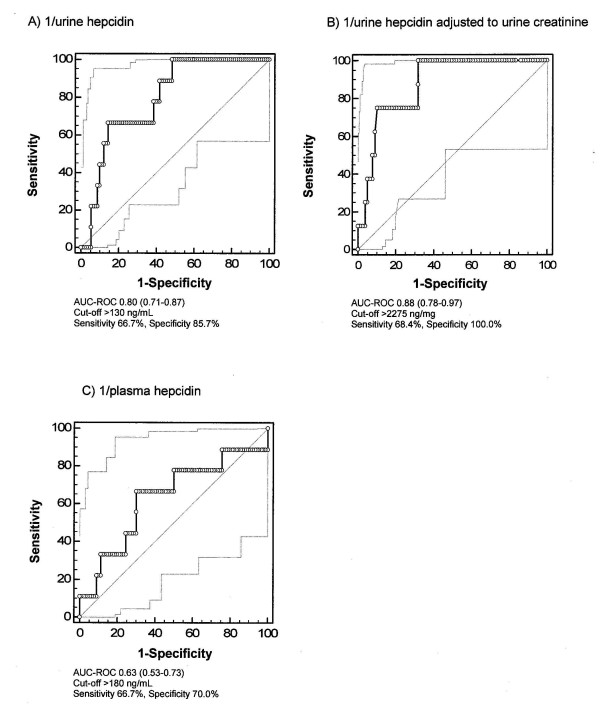
**Predictive indices of hepcidin at six hours after commencement of CPB for not developing AKI**. **(A) **Urine hepcidin. **(B) **Urine hepcidin/urine creatinine. **(C) **Plasma hepcidin. AUC-ROC, area under the curve receiver operating characteristic curve.

**Figure 5:**
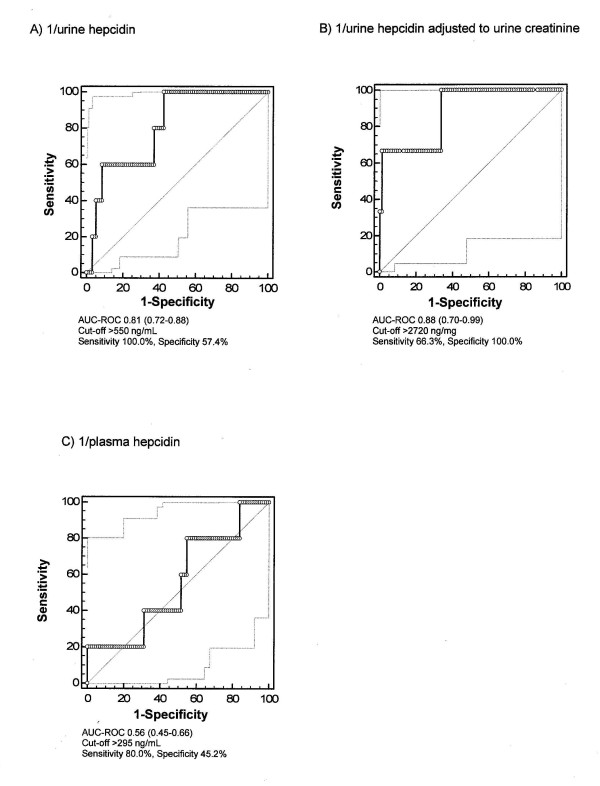
**Predictive indices of hepcidin at six hours for not requiring postoperative renal replacement therapy initiation**. **(A) **Urine hepcidin. **(B) **Urine hepcidin/urine creatinine. **(C) **Plasma hepcidin.

Plasma hepcidin concentrations were three to seven times lower compared to urine hepcidin in patients not developing AKI (Figure 3A vs. Figure 2A). At baseline, patients who did not develop AKI had a mean plasma hepcidin concentration of 112 ng/mL (range, 78 to 203 ng/mL) compared to 55 ng/mL (range, 50 to 146 ng/mL) in patients who developed AKI (Figure 3A). Hepcidin levels at six hours after the start of CPB tripled in patients who developed AKI (167 ng/mL; range, 73 to 325 ng/mL), whereas they only doubled (261 ng/mL; range, 154 to 386 ng/mL) in those who remained AKI-free. Postoperative plasma hepcidin concentration had a lower AUC-ROC curve than (un)adjusted urine hepcidin and was not useful in separating patients with or without AKI (Figures 3A and 4C). The same applied to RRT initiation (Figure 5C).

### Independent predictors of AKI

At six hours after commencement of CPB, urine hepcidin adjusted to urine creatinine (*P *= 0.016) was the only independent predictive biomarker for no AKI (Table 5), and the quality of the model improved considerably (from *R*^2 ^= 0.42 to *R*^2 ^= 0.63). Serum creatinine at six hours after commencement of CPB had no reasonable AUC-ROC (0.69 [95% CI 0.48 to 0.93]) and was not an independent predictor of AKI (*P *> 0.1). Other clinical predictors (model 0) included age, impaired LVEF, COPD and PVD and were retained in the models 1 through 3 after inclusion of renal biomarkers (Table 5).

**Table 5:** Multivariate logistic regression analysis (all patients) of risk factors and biomarkers at six hours after CPB start for the prediction of no AKI^a^

	Model 0 (R^2 ^= 0.42)		Model 1 (R^2 ^= 0.63)		Model 2 (R^2 ^= 0.43)		Model 3(R^2 ^= 0.50)	
Risk factors and biomarkers	Regression coefficient (B) (SE)	*P *value	Regression coefficient (B) (SE)	*P *value	Regression coefficient (B) (SE)	*P *value	Regression coefficient (B) (SE)	*P *value
Age, years	-0.2 (0.1)	0.015	-0.2 (0.1)	0.06	-0.1 (0.1)	0.06	-0.2 (0.1)	0.036
LVEF <35%	-2.9 (1.1)	0.009	-4.1 (1.6)	0.008	-2.7 (1.1)	0.011	-3.0 (1.2)	0.01
COPD	-2.2 (1.0)	0.036	-2.4 (1.3)	0.06	-2.0 (1.0)	0.044	-2.5 (1.1)	0.026
Log urine hepcidin/urine creatinine, ng/mg			2.9 (1.1)	0.011				
Log urine hepcidin, ng/mL					1.2 (0.7)	0.09		
Log plasma hepcidin, ng/mL							2.7 (2.5)	0.56

^a^AKI, acute kidney injury; CPB, cardiopulmonary bypass; LVEF, left ventricular ejection fraction; COPD, chronic obstructive pulmonary disease; SE, standard error. Variables tested on univariate relation with incidence of acute kidney injury (AKI) included all variables displayed in Table 1, type and duration of surgery and renal biomarkers at six hours after start of CPB. Multivariate logistic regression analysis included relevant variables for (no) AKI with univariate *P *value <0.1 (age, atrial fibrillation, LVEF <35%, COPD and peripheral vascular disease). Variables were presented in model 0 if their multivariate *P *value was <0.05 (age, LVEF <35% and COPD). We included each biomarker one after another (models 1 through 3) into multivariate regression analysis to exclude interaction.

### Correlation of urine and plasma hepcidin levels

At baseline, urine hepcidin adjusted to urine creatinine correlated well with plasma hepcidin (*r *= 0.76, *P *< 0.001). We found a good correlation between postoperative plasma hepcidin and urine hepcidin in patients not developing AKI (six hours after CPB: *r *= 0.62, *P *< 0.001), but no correlation in those with subsequent AKI (six hours after CPB: *r *= 0.18, *P *= 0.64).

### Urine/plasma hepcidin ratio and fractional excretion of hepcidin

In AKI-free patients, a urine/plasma hepcidin ratio >1 was observed, which increased from 2.6 to 6.9 over time (Table 3). Patients developing AKI, however, presented with a urine/plasma hepcidin ratio around 1.0, which remained stable over time (Table 3). Overall, the highest FE of hepcidin was detected at six hours after commencement of CPB. However, the FE of hepcidin was about three times higher in those not developing AKI (Table 3).

### Influence of exclusion of patients with preoperative chronic kidney disease

The findings at baseline and interventions and outcomes remained essentially unchanged after patients with preoperative CKD were excluded from the analyses (Tables 1 and 2). The magnitude of urine and plasma hepcidin concentrations and the increase over time or the predictive values of hepcidin did not differ significantly between patients with or without CKD (Table 6). Also, of note, preoperative urine and plasma hepcidin levels or the urine/plasma hepcidin ratio did not correlate with preoperative eGFR (all *r *< 0.2, all *P *> 0.1) (Table 7).

**Table 6:** Predictive values of biomarkers for ruling out AKI excluding patients with preoperative CKD^a^

Biomarkers	AUC-ROC (95% CI)	Sensitivity	Specificity	Cutoff
Urine hepcidin, ng/mL				
6 hours after CPB start	0.81 (0.67 to 0.96)	62.30%	100.00%	>545
24 hours after CPB start	0.83 (0.74 to 0.92)	79.70%	100.00%	>475
Urine hepcidin/urine creatinine, ng/mg				
6 hours after CPB start	0.87 (0.75 to 0.99)	70.00%	100.00%	>2820
24 hours after CPB start	0.75 (0.57 to 0.94)	76.50%	80.00%	>1,762
Plasma hepcidin, ng/mL				
6 hours after CPB start	0.70 (0.46 to 0.98)	69.00%	80.00%	>175
24 hours after CPB start	0.54 (0.23 to 0.86)	N/A	N/A	N/A

^a^AKI, acute kidney injury; AUC, area under the curve; ROC, receiver operating characteristic; CKD, chronic kidney disease; CPB; cardiopulmonary bypass; N/A, values not presented for AUC-ROC <0.6. AKI is defined as Risk, Injury, Failure, Loss, End-stage renal disease classification of AKI (RIFLE) class R or worse including serum creatinine increase and urine output decrease.

**Table 7:** Correlation coefficients of hepcidin with preoperative eGFR^a^

Baseline variables	Spearman's ρ	Preoperative eGFR
Urine hepcidin	Correlation coefficient	0.15
	Significance (two-sided)	0.15
	Number of patients	100
Urine hepcidin adjusted to urine creatinine	Correlation coefficient	0.07
	Significance (two-sided)	0.51
	Number of patients	100
Plasma hepcidin	Correlation coefficient	0.043
	Significance (two-sided)	0.67
	Number of patients	100
Urine/plasma hepcidin ratio	Correlation coefficient	0.16
	Significance (two-sided)	0.11
	Number of patients	100

^a^eGFR, estimated glomerular filtration rate.

## Discussion

### Key findings

In a control cohort recruited to participate in an interventional study of AKI after CPB, we found that higher urine hepcidin concentrations determined early postoperatively were associated with ruling out AKI, whereas postoperative hepcidin remained at preoperative levels in patients with subsequent AKI. Furthermore, early postoperative urine hepcidin levels and, even more so, urine hepcidin adjusted to urine creatinine discriminated patients not developing AKI or not requiring RRT initiation from those who did, although the event rate was low. Early postoperative urine hepcidin levels adjusted to urine creatinine were also independent predictors of ruling out AKI. On the other hand, plasma hepcidin levels and serum creatinine determined early postoperatively were not associated with not developing AKI after cardiac surgery. The study findings remained essentially unchanged after excluding patients with preoperative CKD. Preoperative values of urine hepcidin correlated with plasma hepcidin levels; however, postoperatively, this correlation was present only in patients not developing AKI. In patients with AKI, postoperative urine hepcidin concentrations were three to seven times lower than those in patients without AKI and similar to plasma hepcidin levels.

### Relation to previous studies

The incidence of AKI in our patient cohort was similar to that recently described by Argalious *et al. *[24] but lower than that previously reported by Haase *et al. *[25] in an Australian cohort. A multitude of studies have investigated hepcidin in a number of nonrenal diseases, for example, as a marker of anemia [26-28], but also in CKD [29,30]. Hepcidin is a peptide hormone synthesized in hepatocytes and is detected in the normal kidney, heart and brain [31,32]. Hepcidin is centrally involved in iron homeostasis, and its synthesis is increased under inflammatory conditions via interleukin 6. On the other hand, its synthesis is reduced in the setting of hypoxia [11,26,28,33,34].

In the kidney, systemic hepcidin is filtered through the glomerulus, partly reabsorbed into the proximal tubules and converted to active hepcidin-25 *in situ *[14]. Within the kidney, hepcidin is expressed in the apical tubular epithelium of the thick ascending limb of the loop of Henle [13]. Preoperative serum hepcidin concentrations were similar to those reported in five cardiac surgery patients [35] as well as in healthy adults [20]. Also, in a previous study, urine hepcidin levels adjusted to urine creatinine and plasma hepcidin concentrations correlated well in healthy individuals [20], as seen preoperatively in our cohort.

Using mass spectroscopy, Ho *et al. *[14] detected a urine hepcidin peak with increased frequency and intensity on the first postoperative day in patients with a postoperative serum creatinine increase <10% from baseline to peak compared to patients with AKI. This hypothesis-generating approach maximized the likelihood of detecting a signal but may not be generalized to consecutive patients. Also, hepcidin assays based on mass spectroscopy techniques [36] are semiquantitative and depend on expensive equipment that is not widely available. Therefore, the authors suggested that quantification of hepcidin in both plasma and urine samples in consecutive patients would be necessary to elucidate the potential diagnostic and prognostic value of hepcidin for AKI after cardiopulmonary bypass [37]. None of the studies reported the quantitative measurement of plasma and urine hepcidin levels as renal biomarkers in patients at risk of AKI after CPB. Our findings are consistent with those reported by Ho *et al. *[14]. By contrast, our results deviate from those observed in a small patient cohort [38] describing no association between postoperative fractional excretion of hepcidin and AKI on the first postoperative day.

### Significance of study findings

As a low-molecular-weight peptide (2.78 kDa), hepcidin would be expected to pass unimpeded into the glomerular ultrafiltrate with a sieving coefficient of 1. However, at baseline, we found a low fractional excretion of hepcidin, implying significant tubular reabsorption and/or catabolism of hepcidin along the nephron. Markedly increased postoperative fractional excretion of hepcidin (10-fold) in patients not developing AKI may point toward (1) relatively preserved filtration, (2) reduced or saturated reabsorption, (3) increased active secretion and (4) increased local synthesis of hepcidin along the nephron [13], or any combination of these factors. Many studies [39-41] have suggested that there is tubular injury with CPB [3]. Thus one might expect that reduced hepcidin absorption alone would be greater in patients who subsequently develop AKI, resulting in higher urine hepcidin concentrations. The fact that the opposite occurs makes this mechanism less likely. As active hepcidin secretion has not been demonstrated previously, this mechanism also seems less likely. Relatively preserved filtration in the setting of a simultaneous decrease in reabsorption, however, seems possible and more likely. Decreased reabsorption would explain an increase in urine hepcidin levels for all patients. Later on, relatively preserved filtration (reflecting better GFR) would be expected to further elevate urine hepcidin concentrations in recovering patients and thereby identify those at lower risk of subsequent AKI.

Loss of integrity of tubular tight junctions with tubular backleak of urine hepcidin would explain a postoperative increase in plasma concentrations in patients developing AKI, but not its observed low magnitude. Also, one would expect hepcidin and creatinine to backleak equally, unless hepcidin was then sequestered or less was delivered downstream. Finally, the additional role of local hepcidin release remains unknown, and upregulation of tubular hepcidin [13], as is the case for lipocalin 2 [42], may be an adaptive response to injury. Hepcidin-mediated intracellular iron sequestration may act to limit ischemia-reperfusion injury in a manner analogous to exogenous deferoxamine administration [14]. Irrespective of the underlying mechanisms, measurement of urine hepcidin concentrations, combined with measurements of one or more other biomarkers (that is, lipocalin 2), may provide additional information for the early identification of individuals who will or will not develop AKI.

### Strengths and limitations

To the best of our knowledge, this study is the first to describe in detail the relationship between urine and plasma hepcidin and AKI in humans after CPB. We used a novel competitive enzyme-linked immunoassay for human hepcidin [20] which accurately and reproducibly detects physiologic and pathologic changes in plasma or urine hepcidin levels. We simultaneously measured both plasma and urine hepcidin levels to elucidate whether there is an interrelationship between them.

However, we focused on a single renal biomarker (hepcidin) involved in complex traits of iron metabolism. Our findings are limited to a single-center study, and center-specific effects cannot be excluded. Although in our study the event rate was low, the findings were consistent with those of a previous publication [14] and with those of another independent observational study [43]. Our study appears to be underpowered for the detection of a preoperative difference in hepcidin levels. AKI after CPB has specific etiopathologic factors, and these findings may not be generalized to AKI in other contexts. Furthermore, proof of association does not imply that hepcidin is causatively involved in the pathogenesis of or response to AKI after CPB.

### Future studies

Our study findings suggest the need to investigate the role and source of urine hepcidin in the pathophysiology of AKI after cardiac surgery using CPB. Such investigations may encompass interventions such as the modulation of iron release and disposal during CPB. In addition, it now seems desirable to investigate the diagnostic and prognostic values of hepcidin in other forms of AKI as well as the combined diagnostic and prognostic value of hepcidin in conjunction with other biomarkers.

## Conclusions

Our findings suggest that early postoperative urine hepcidin determination has additive value in ruling out AKI after CPB, thereby potentially helping to triage patients. The nature of this association, the central biological role of hepcidin in iron metabolism and the possible importance of iron in the pathogenesis of CPB-associated AKI, taken together, identify hepcidin as an interesting subject for future research in this context.

## Key messages

• Cardiopulmonary bypass increases plasma and urine hepcidin levels.

• The increase in urinary hepcidin levels is greater in patients who do not develop AKI.

• Urine hepcidin has additive value in ruling out acute kidney injury in patients after cardiac surgery.

• Urine hepcidin is predictive of not needing postoperative RRT.

• Plasma hepcidin does not rule out the development of acute kidney injury.

## Abbreviations

AKI: acute kidney injury, AUC: area under the curve; CKD: chronic kidney disease; CPB: cardiopulmonary bypass; FE: fractional excretion; RIFLE: Risk, Injury, Failure, Loss, End-stage renal disease classification of AKI; RRT: renal replacement therapy; ROC: receiver operating characteristic.

## Competing interests

MW is a shareholder, president and chief executive officer of Intrinsic LifeSciences, a developer and distributor of an enzyme-linked immunosorbent assay for hepcidin. MW has received consulting fees and grant support from Centocor-Ortho Research and Development. JRP and RB are named in a US preliminary patent application in conjunction with MW. RB has received consulting fees from Gambro, Biosite, Abbott Diagnostics and Philips Medical Systems, as well as grant support from Fresenius Kabi, Bard, Pfizer and Gambro. MH received lecture fees from Abbott Diagnostics and Biosite/Alere.

## Authors' contributions

MH, AHF and RB conceived of the study, participated in its design and drafted the manuscript. MW and VO carried out the hepcidin measurements and, together with JRP, MP, HK, RH and PRM, participated in its design, helped to draft the manuscript and provided critical intellectual content. All authors read and approved the final manuscript.
